# GelMA-Based Hydrogels for Controlled Delivery of Antimicrobial Peptides against *Candida albicans* Biofilm

**DOI:** 10.1021/acsomega.6c06773

**Published:** 2026-08-14

**Authors:** Beatriz Ribeiro Ribas, Xavier L. Tabil, Xiongbiao Chen, Walter Luiz Siqueira, Eduardo Buozi Moffa, Janaina Habib Jorge

**Affiliations:** † Department of Dental Materials and Prosthodontics, School of Dentistry, São Paulo State University, Araraquara, São Paulo 4800-901, Brazil; ‡ College of Engineering, 7235 University of Saskatchewan, Saskatoon, Saskatchewan S7N 5A2, Canada; § College of Dentistry, University of Saskatchewan, Saskatoon, Saskatchewan S7N 5A2, Canada; ∥ Department of Dental Materials and Prosthodontics, Associated Colleges of Education (FAE), University Center, São João da Boa Vista, São Paulo 13870-377, Brazil

## Abstract

Objective: This study aimed to engineer and characterize GelMA hydrogels and dual-cross-linked GelMA–alginate hydrogels as tunable delivery platforms for antimicrobial peptides (AMPs; Histatin-5, GK-17, and INLK), and to evaluate their physicochemical properties, release kinetics, cytocompatibility, and antibiofilm efficacy against *Candida albicans*. Methods: Hydrogels with varying GelMA concentrations (5%, 7.5%, and 10%), with or without alginate, were fabricated and characterized in terms of microstructure, rheological and mechanical properties, swelling, degradation, and AMP release kinetics. Cytocompatibility was assessed using human gingival fibroblasts, and antimicrobial activity was evaluated through colony-forming unit counts, metabolic activity assays, biomass quantification, and confocal microscopy. Results: All hydrogels exhibited suitable moldability, pseudoplastic behavior, and structural stability. Increasing GelMA concentration and alginate incorporation enhanced mechanical properties: G10 displayed a higher compressive modulus (12,437.39 ± 565.33 Pa; *p* < 0.0001) compared with the other GelMA-only groups, while G7.5A showed the highest value overall (18,090.45 ± 2324.12 Pa; *p* < 0.0001). Sustained peptide release was observed over 48 h, with faster release from lower GelMA concentrations; G5 released 20.46% of Histatin-5-FAM after 48 h (*p* < 0.006). All formulations showed high cytocompatibility, with viability exceeding 100% at 24 h, indicating a stimulatory effect on fibroblast metabolic activity rather than mere absence of cytotoxicity. Hydrogels loaded with INLK or GK-17 significantly reduced *C. albicans* biofilm viability, biomass, and metabolic activity, whereas unloaded hydrogels showed no antifungal effect. G5-INLK reduced biofilm viability by approximately 1.6 log (*p* < 0.006) among the GelMA 5% groups, G7.5-INLK and G7.5-GK-17 reduced it by approximately 2 log (*p* < 0.0001), and G10-INLK produced a 0.9–1.2 log reduction. Conclusion: GelMA-based hydrogels provide a tunable and biocompatible platform for controlled AMP delivery, enabling effective disruption of *C. albicans* biofilms. This approach represents a promising strategy for the localized treatment of *C. albicans*-associated oral biofilm infections.

## Graphical abstract

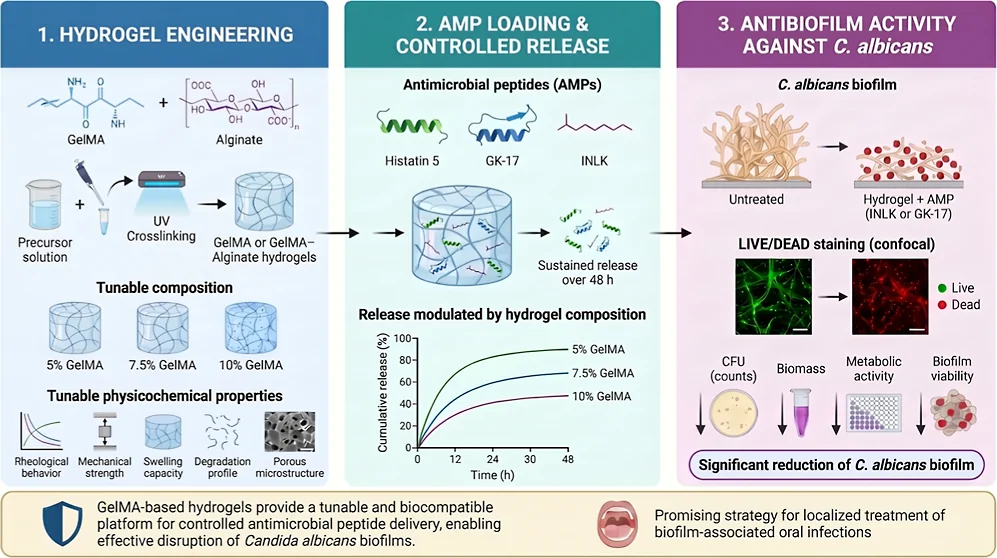

## 1 Introduction

The oral cavity harbors a diverse range of microorganisms, including bacterial and fungal species, which colonize both soft and hard tissues as structured biofilms or exist as planktonic cells in saliva.^1^ Under healthy conditions, this microbiota remains in a stable, commensal state; however, disruptions in this balance may lead to disease.^2^ Among the fungal species, *Candida albicans* is the most frequently associated with oral infections and commonly coexists with bacteria in polymicrobial biofilms.^2–5^ The organization of microorganisms into biofilms is a key virulence factor, as the formation of a self-produced extracellular polymeric substance (EPS) matrix creates a protective microenvironment that limits antimicrobial penetration, reduces bioavailability, and contributes to treatment resistance, further aggravated by salivary clearance.^6^

The rise of antifungal resistance, particularly in biofilm-associated infections, reinforces the urgent need for alternative treatment approaches.^7,8^ Antimicrobial peptides (AMPs) have emerged as promising candidates owing to their broad-spectrum activity and low propensity to induce resistance.^9^ These small, predominantly cationic molecules exert their effects through membrane disruption and intracellular targeting, while also modulating immune responses by recruiting neutrophils and monocytes.^10,11^ Histatin-5 is one of the most extensively characterized salivary antimicrobial peptides, exhibiting potent activity against *C. albicans* through intracellular targeting and disruption of mitochondrial function, while displaying minimal toxicity toward host cells.^12–14^ GK-17, a synthetic 17-amino acid peptide derived from human cathelicidin LL-37, has demonstrated potent and rapid anti-*C. albicans* activity, including efficacy against biofilms, even in the presence of biological matrices such as saliva and serum.^16^ Notably, GK-17 exhibited a lower propensity for resistance development compared to nystatin, along with favorable biocompatibility, supporting its potential as an antifungal candidate.^16^ INLK, a synthetic peptide derived from wasp venom, rationally designed to enhance antimicrobial efficacy and stability, has also shown promising antifungal activity against *C. albicans*.^15,17^ Together, these three peptides represent distinct structural classes and mechanisms of action, enabling the evaluation of complementary antimicrobial strategies within the same hydrogel-based delivery platform.

Despite these advantages, AMPs face major limitations, including proteolytic and hydrolytic degradation and reduced stability in physiological environments. Furthermore, their cationic nature promotes interactions with anionic serum proteins, thereby reducing antimicrobial efficacy.^9^ To address these limitations, advanced delivery systems, such as gelatin methacrylate (GelMA) hydrogels, have emerged as promising platforms for the controlled delivery of bioactive molecules. GelMA is a biocompatible, photo-cross-linkable hydrogel with inherent mucoadhesive and tissue-regenerative properties, making it notably suitable for oral applications.^18,19^ Importantly, the physicochemical properties of GelMA can be finely tuned by modulating parameters such as the degree of methacrylation, polymer concentration, photoinitiator content, and cross-linking conditions, as well as by incorporating functional additives. These modifications directly influence the network architecture, including pore size, cross-linking density, and degradation behavior, which in turn govern the diffusion and controlled release of encapsulated molecules.^20^ Such tunability enables precise regulation of the stability and bioavailability of therapeutic agents. Accordingly, GelMA-based systems have been successfully employed for the delivery of antimicrobial peptides in applications such as diabetic wounds, keratitis, and skin injuries, demonstrating enhanced peptide stability and improved treatment outcomes.^21–24^

Although GelMA hydrogels offer several advantages, their mechanical properties may be limited by their relatively low viscosity and structural stability, which can restrict their use in load-bearing or dynamic environments. One strategy to overcome these limitations involves incorporating secondary polymer networks, such as alginate. Alginate is a naturally derived, biocompatible polysaccharide that is widely used in biomedical applications because of its mild gelation via ionic cross-linking and ease of processing.^18,19,25^ Therefore, the development of hybrid hydrogels combining GelMA and alginate has gained increasing attention. This combination enables the formation of dual-cross-linked networks that integrate covalent (GelMA) and ionic (alginate) interactions, thereby enhancing mechanical strength, improving viscoelastic behavior, and modulating network architecture.^26^ Importantly, these structural features influence porosity and diffusion properties, thereby supporting the controlled delivery of bioactive molecules, such as antimicrobial peptides.^19,27,28^

Given the need for effective localized strategies against biofilm-associated oral infections, this study developed and characterized GelMA and dual-cross-linked GelMA–alginate hydrogels as tunable platforms for the controlled delivery of antimicrobial peptides (histatin 5, GK-17, and INLK), with systematic evaluation of their physicochemical properties, release kinetics, biocompatibility, and antibiofilm efficacy against *C. albicans*.

## 2 Material and Methods

### 2.1 Preparation of GelMA Hydrogel

GelMA was synthesized from porcine type A gelatin (Sigma-Aldrich, USA) following previously established protocols.^29^ Briefly, gelatin was dissolved in PBS at 50 °C (10% w/v), followed by dropwise addition of methacrylic anhydride under constant stirring for 2 h. The reaction mixture was diluted with PBS, dialyzed against distilled water for 5 days (with daily water changes), filtered (0.22 μm), freeze-dried, and stored at −20 °C until use.

### 2.2 Formulations of GelMA Hydrogel

GelMA hydrogels (5%, 7.5%, and 10% w/v) were prepared with 0.025% (w/v) LAP (lithium phenyl-2,4,6-trimethylbenzoylphosphinate; Sigma-Aldrich, USA) and photocured for 20 s using a blue LED light source (385–515 nm; VALO Grand, Ultradent, USA). For hybrid formulations, alginate (2% w/v) was incorporated into the prepolymer solution. Following photo-cross-linking, the samples were immersed in a CaCl_2_ (100 mM) solution for 2 min to induce ionic cross-linking. GelMA hydrogels were denoted as G5, G7.5, and G10, while GelMA–alginate formulations were denoted as GA5, GA7.5, and GA10.

### 2.3 Physical Characterization of Samples

#### 2.3.1 Scanning Electron Microscopy (SEM)

The microstructural morphology of the hydrogels was analyzed using scanning electron microscopy (SEM; Phenom, FEI Company, Hillsboro, OR, USA) at an accelerating voltage of 10–15 kV. GelMA and GelMA–alginate samples were frozen at −80 °C, freeze-dried overnight (−50 °C), and sputter-coated with gold prior to imaging. Six images per sample were acquired from different regions at magnifications of up to 500×.

#### 2.3.2 Degradation and Swelling Ratios

The degradation and swelling behavior of the hydrogels were evaluated by incubating the samples in phosphate-buffered saline (PBS) at 37 °C under orbital shaking (76 rpm). The initial wet weight (*W*_0_) was recorded using a precision balance (ME203E, Mettler Toledo, Switzerland). Samples were weighed at predetermined time points (1, 3, 6, 16, 24, and 48 h). The degradation ratio (DR, %) was calculated as DR = [(*W*_0_ – *W*_t_)/*W*_0_] × 100, and the swelling ratio (SR, %) was calculated as SR = [(*W*_t_ – *W*_0_)/*W*_0_] × 100.

#### 2.3.3 Rheology

Rheological characterization was performed using a Discovery Hybrid Rheometer 20 (HR20; TA Instruments, USA) with a cone-and-plate configuration, a 51 μm truncated gap, and a solvent trap to prevent the samples from drying. Approximately 1 mL of each GelMA formulation in the liquid phase was loaded and trimmed on the testing stage. To record the viscosity profiles of the GelMA formulations, the samples were sheared at a logarithmic rate of 0.1–100 s-1 at 37 °C. In addition, the temperature dependence of the viscosity was characterized by decreasing the temperature from 25 to 50 °C at a rate of 5 °C/min. A constant shear rate of 1 s^–1^ was applied for the duration of the experiment.

#### 2.3.4 Compression

Samples were gently blot-dried to remove excess surface moisture and imaged from above to determine the cross-sectional area. Hydrogels were placed between nonporous compression platens, and the initial height was recorded. Unconfined compression tests were performed at room temperature using a displacement rate of 1 mm/min (ElectroForce Biodynamic 5115, TA Instruments, USA) until 30% strain was reached. The compressive modulus was calculated from the linear region of the stress–strain curve.

### 2.4 Cytocompatibility of GelMA Formulations

Human gingival fibroblasts (HGFs; ATCC, catalog no. PCS-201–018) were cultured in Dulbecco’s Modified Eagle Medium (DMEM; Gibco, USA) supplemented with 10% fetal bovine serum (FBS; Gibco, USA) and 1% antibiotic solution (Sigma-Aldrich, USA) under standard conditions (37 °C, 5% CO_2_). As HGFs constitute a commercially sourced, standardized cell line rather than primary cells isolated in-house, no additional institutional ethical approval was required for their use in this study. The cells were subcultured until they reached confluence, detached using 0.25% trypsin, and resuspended in fresh medium.

Hydrogels were placed in 48-well plates, and cells were seeded at a density of 2 × 10^4^ cells per well. Control groups included cells cultured without hydrogels (live control), cells treated with 0.1% Triton X-100 (dead control), and hydrogels without cells (background control).

After 24 h of incubation, the medium was replaced with DMEM containing 10% Alamar Blue reagent (Invitrogen, Thermo Fisher Scientific, USA), and the plates were incubated under standard conditions protected from light. Fluorescence was measured using a microplate reader (Synergy, BioTek, USA) at excitation/emission wavelengths of 560/590 nm after 4, 6, and 24 h of incubation. The experiments were performed in triplicate across three independent assays (*n* = 9).

### 2.5 Preparation of AMP-Loaded GelMA Hydrogels

The following antimicrobial peptides were used: histatin-5 (Hst-5), GK-17, and INLK. Peptides were homogeneously incorporated into the GelMA prepolymer solution at a concentration of 100 μg per sample prior to cross-linking. The mixtures were then subjected to photo- and ionic cross-linking, as previously described, enabling peptide entrapment within the hydrogel network. The resulting peptide concentrations were 329 μM (Hst-5), 466 μM (GK-17), and 661 μM (INLK), based on the molecular weight of each peptide.

#### 2.5.1 Release Kinetics of Histatin 5-FAM from GelMA Samples

Hst-5 labeled with 5-carboxyfluorescein (FAM) was used to evaluate peptide release from the different GelMA formulations as a representative AMP model, given that fluorescently labeled analogs of GK-17 and INLK were not available within the time frame of this study. As all three peptides share a cationic nature and comparable molecular weights, Hst-5–FAM was considered a reasonable surrogate for estimating general release behavior across formulations. Briefly, 100 μg of Hst-5–FAM was incorporated into each prepolymer solution under light-protected conditions. Following photo-cross-linking, hydrogels were transferred to 24-well plates, and 1 mL of phosphate-buffered saline (PBS) was added to each well. Samples were incubated at 37 °C under orbital shaking (76 rpm) and protected from light.

At predetermined time points (1, 3, 6, 16, 24, and 48 h), 100 μL aliquots of the release medium were collected and transferred to 96-well plates for fluorescence quantification using a microplate reader (Synergy, BioTek, USA) at excitation and emission wavelengths of 495/520 nm. The collected volume was not replaced during the experiments. The peptide concentration was determined using a standard calibration curve, and the cumulative release was calculated relative to the initial peptide loading. The experiments were performed in triplicate across three independent assays (*n* = 9).

### 2.6 Antimicrobial Activity of GelMA Hydrogel Loaded with AMPs

The antimicrobial activity of GelMA and GelMA–alginate hydrogels loaded with AMPs against *C. albicans* biofilms was evaluated using colony-forming unit (CFU) assays, metabolic activity assessment (Alamar Blue), and total biofilm biomass quantification (crystal violet staining). In addition, analysis of *C. albicans* cell viability by Confocal Laser Scanning Microscopy (CLSM) was performed.

#### 2.6.1 *C. albicans* Biofilm Formation and Treatment Protocol

*C. albicans* (SC5314) were stored at −80 °C. Prior to experiments, cells were thawed and streaked onto Sabouraud dextrose agar supplemented with chloramphenicol (SDA; Acumedia, USA), followed by incubation at 37 °C for 48 h. A preinoculum was prepared in yeast nitrogen base (YNB) and incubated overnight (16 h) at 37 °C.

The preinoculum was diluted (1:20) in fresh YNB and incubated for 8 h to reach the mid log phase. Cell density was adjusted to 1 × 10^6^ cells/mL based on optical density measurements at 540 nm. Cells were then centrifuged, washed twice with PBS, and resuspended in RPMI-1640 medium.

Aliquots of the *C. albicans* suspension were added to 48-well plates and incubated at 37 °C under orbital shaking (76 rpm) for 90 min to allow initial adhesion. Wells were subsequently washed with PBS, and fresh RPMI medium was added for biofilm development over 24 h.

After biofilm formation, GelMA hydrogels containing AMPs were applied according to the experimental groups, with peptide-free hydrogels used as controls. Samples were incubated at 37 °C under orbital shaking (76 rpm) for 16 h.

#### 2.6.2 *C. albicans* Biofilm Colony-Forming Unit (CFU/mL) Assay

After treatment, the hydrogels were removed, and the wells were washed twice with PBS. Biofilms were mechanically disrupted by scraping, and the resulting suspensions were collected in PBS. Serial dilutions (10^–1^ to 10^–5^) were prepared, and 10 μL of each dilution was plated in duplicate on Sabouraud dextrose agar (SDA; Acumedia, USA). The plates were incubated at 37 °C for 48 h, and colony-forming units (CFU/mL) were determined. The CFU/mL values were calculated based on the dilution factor. Experiments were performed in triplicate across three independent assays (*n* = 9).

#### 2.6.3 Metabolic Activity of *C. albicans* Cells through Alamar Blue Assay

After treatment, the wells were washed twice with PBS, and RPMI-1640 medium containing 10% Alamar Blue reagent was added to each well. The plates were covered with aluminum foil to protect them from light and incubated at 37 °C under orbital shaking (76 rpm) for 4 h. Subsequently, 200 μL from each well was transferred to a 96-well plate, and fluorescence was measured using a Synergy microplate reader at an excitation wavelength of 560 nm and an emission wavelength of 590 nm. Cell viability was expressed as the percentage of the untreated control by normalizing the relative fluorescence unit (RFU) values of each sample to the mean RFU of the untreated control group, which was considered 100% viability (% viability = RFU treated/RFU untreated control × 100). The procedures were performed in triplicate on three separate occasions (*n* = 9).

#### 2.6.4 Quantification of Total *C. albicans* Biofilm Biomass by Crystal Violet Staining

After treatment, the wells were washed twice with PBS and air-dried at 35 °C for 20 min. Biofilms were fixed with methanol for 15 min, washed with PBS, and stained with 0.1% crystal violet for 5 min. Excess stain was removed by washing with PBS, and the retained dye was solubilized using 99% ethanol. Aliquots (100 μL) were transferred to 96-well plates, and the absorbance was measured at 595 nm using a microplate reader (Synergy, BioTek, USA). The experiments were performed in triplicate across three independent assays (*n* = 9).

#### 2.6.5 Analysis of *C. albicans* Cell Viability by Confocal Laser Scanning Microscopy (CLSM)

After treatment, the wells were washed twice with PBS and stained with a PBS solution containing SYTO-9 and propidium iodide (PI) (1 μL of each dye per 1 mL of PBS). Samples were incubated at 37 °C for 30 min, protected from light. After staining, the wells were washed with PBS to remove excess dye.

Images were acquired using a confocal laser scanning microscope (LSM 900 with Airyscan 2, Carl Zeiss, Germany) equipped with a 10× objective. Live cells were stained green (SYTO-9) and dead cells red (PI). The excitation/emission wavelengths were approximately 480/500 nm for SYTO-9 and 490/635 nm for PI.

### 2.7 Statistical Analysis

Descriptive statistics were calculated for each variable. Data were assessed for outliers, normality (Shapiro–Wilk test), and homoscedasticity (Levene’s test). Statistical analyses were performed using GraphPad Prism 8 (GraphPad Software, USA), including repeated-measures ANOVA, two-way ANOVA, and three-way ANOVA, followed by Tukey’s post hoc tests. The significance level was set at α = 0.05.

## 3 Results

### 3.1 Preparation and Characterization of GelMA Hydrogels

All hydrogel formulations exhibited good moldability. Scanning electron microscopy (SEM) images (Figure 1) revealed that increasing GelMA concentration resulted in a more organized and dense network structure. G5 samples displayed a highly porous architecture, whereas the G7.5 and G10 formulations maintained porosity but exhibited reduced pore size and a more uniform pore distribution. The incorporation of alginate (2%) did not result in noticeable changes in microstructural features. These findings suggest an increase in cross-linking density with higher GelMA concentrations.

**Figure 1:**
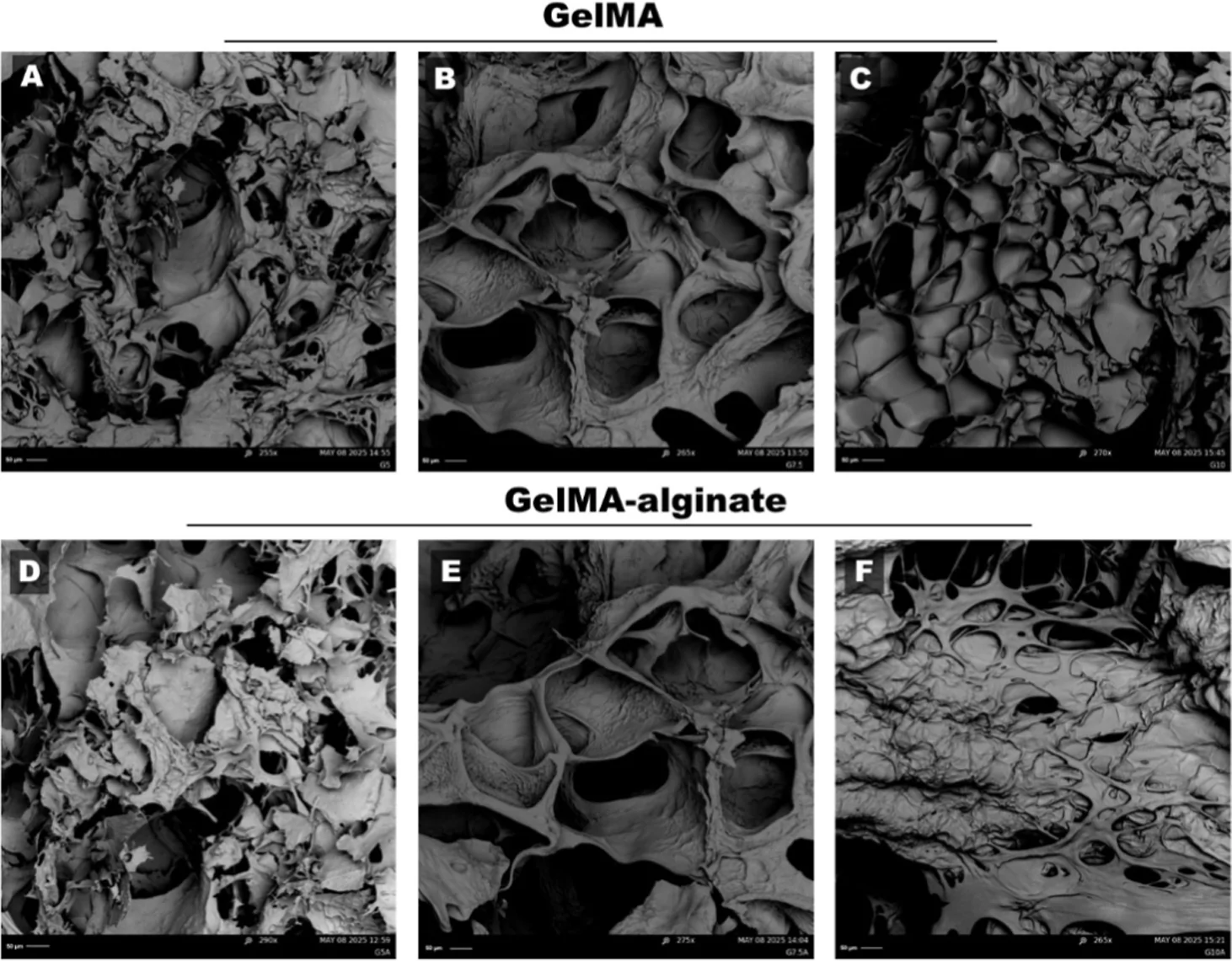
Scanning electron microscopy (SEM) images of GelMA-based hydrogels with and without alginate incorporation. (A–C) GelMA at 5%, 7.5%, and 10% (w/v), respectively. (D–F) GelMA (5%, 7.5%, and 10% w/v) combined with alginate. Samples were freeze-dried and sputter-coated with gold prior to imaging. The images illustrate variations in surface morphology and network organization associated with polymer concentration and alginate addition.

### 3.2 Mechanical Characteristics of GelMA Hydrogels

The rheological behavior of GelMA formulations was evaluated to compare the mechanical properties of the hydrogels. As shown in Figure 2A, viscosity decreased with increasing shear rate (0.1–100 s^–1^), indicating non-Newtonian pseudoplastic (shear-thinning) behavior. This rheological profile is consistent with desirable handling properties, such as injectability and spreadability. Flow curves fitted to the power law model yielded a flow behavior index (*n*) < 1 for all formulations, confirming their pseudoplastic nature.

**Figure 2:**
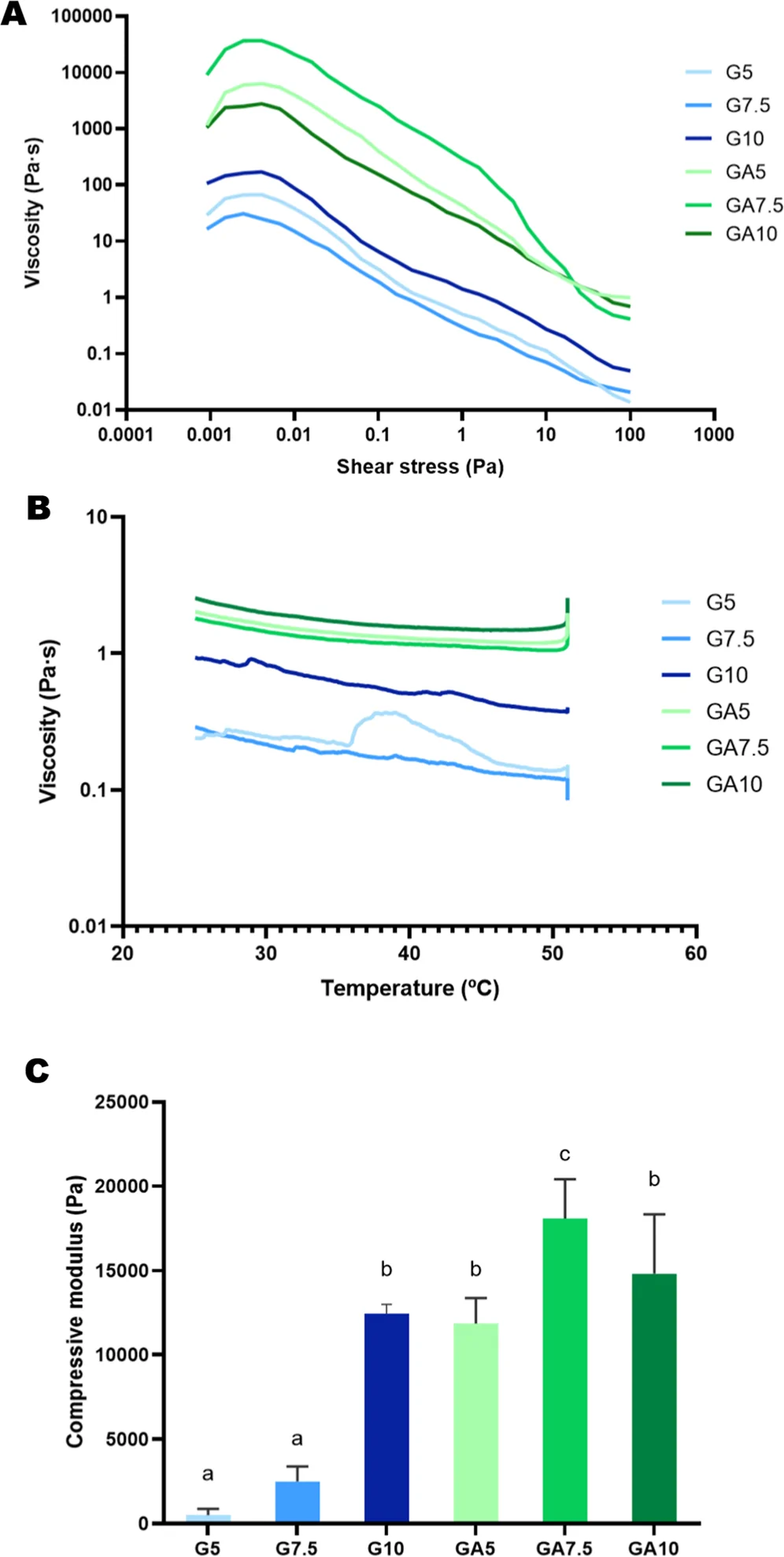
Rheological and mechanical characterization of GelMA-based hydrogel formulations. (A) Flow curves showing the relationship between viscosity (Pa·s) and shear stress (Pa). Samples were subjected to increasing shear stress at 25 °C. (B) Temperature-dependent viscosity profile obtained under a constant shear rate of 10 s^–1^ during a temperature ramp from 25 to 50 °C. (C) Compressive modulus (Pa) determined by unconfined compression testing at a displacement rate of 1 mm/min. Data are presented as mean ± standard deviation (*n* = 3). Logarithmic scales were applied where appropriate to highlight shear-thinning behavior. Statistical analysis was performed using two-way ANOVA followed by Tukey’s post hoc test, with significance set at *p* < 0.05. Different letters indicate statistically significant differences among groups.

The temperature-dependent viscosity profile (Figure 2B) showed a slight decrease in viscosity with increasing temperature (25–50 °C) in all groups, indicating thermal softening. No abrupt sol–gel transition was observed within this range, suggesting sufficient thermal stability.

The compressive modulus (Figure 2C) increased with higher GelMA concentrations and the incorporation of alginate (*p* < 0.0001). Among GelMA formulations, G10 exhibited the highest modulus (*p* < 0.0001), with no significant difference compared to GA10 (*p* = 0.09). The GA7.5 group showed a significantly higher modulus than GA5 and GA10 (*p* < 0.0001). These findings indicate that both polymer concentration and dual cross-linking contribute to mechanical reinforcement of the hydrogel network.

### 3.3 Biodegradation Behavior of GelMA Hydrogels on PBS

Additionally, the swelling behavior of the GelMA formulations was evaluated (Figure 3B,D). The GA7.5 group exhibited the highest swelling ratio (83.44%) during the first 3 h of incubation and was significantly different (*p* < 0.0001) from the other groups. After 6 h of incubation, all groups began to lose mass and showed a decrease in swelling ratio. By 48 h, all formulations presented a similar swelling profile, except GA5, which displayed a significantly lower swelling ratio compared to both G5 and GA7.5 (*p* < 0.0001), consistent with the results observed in the degradation assay.

**Figure 3:**
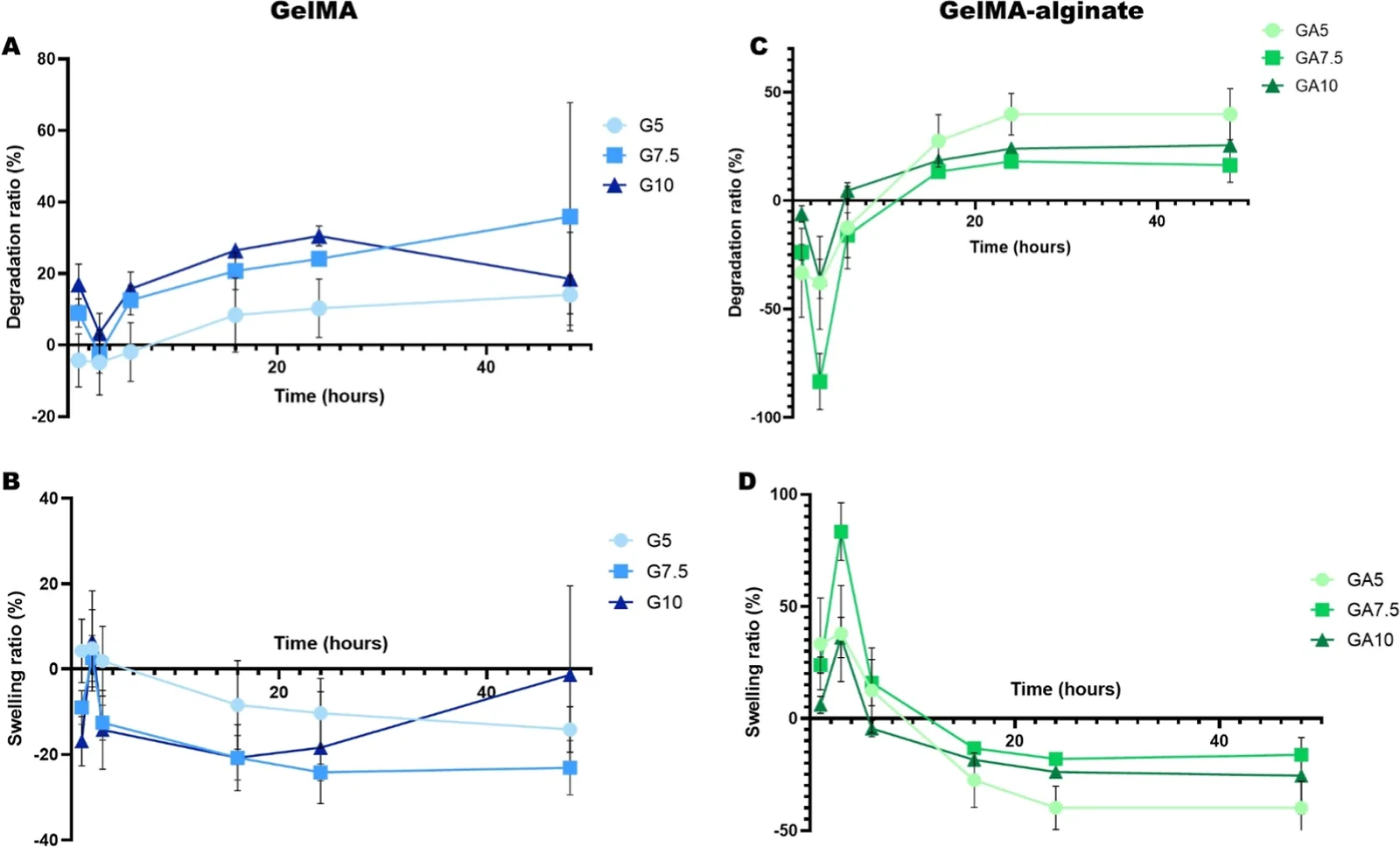
Degradation and swelling behavior of GelMA-based hydrogel formulations after 48 h of incubation in PBS at 37 °C. (A, C) Degradation ratio (%) of GelMA (A) and GelMA-alginate (C) hydrogels. (B, D) Swelling ratio (%) of GelMA (B) and GelMA-based (D) hydrogels. Formulations included GelMA at 5%, 7.5%, and 10% (w/v). Measurements were performed at predetermined time points (0, 1, 3, 6, 16, 24, and 48 h). Data are presented as mean ± standard deviation (*n* = 9).

At the end of 48 h, the remaining masses were as follows: 0.079 ± 0.002 (G5), 0.071 ± 0.002 (G7.5), 0.076 ± 0.006 (G10), 0.052 ± 0.009 (GA5), 0.085 ± 0.006 (GA7.5), and 0.069 ± 0.009 (GA10). The incorporation of alginate into the GelMA hydrogels influenced the degradation rate (Figure 3A,C). After 24 h, the GA5 group exhibited a significant mass loss compared to earlier time points. The GA7.5 and GA10 formulations began to lose mass after 6 h; degradation reached a plateau at 24 h for GA7.5 and at 16 h for GA10. Nonetheless, these reductions were statistically significant compared to the previous time points. After 48 h, all formulations showed a similar degradation profile, except for GA5, which displayed significantly greater degradation compared to both G5 and GA7.5 (*p* < 0.0001).

### 3.4 Drug Release Kinetics of GelMA Hydrogels Loaded with Histatin 5-FAM over 48 h

The release profile of histatin 5–FAM from the GelMA formulations is presented in Figure 4. An initial increase in peptide release was observed, reaching a peak at 6 h, followed by a sustained release phase up to 48 h. Among all the formulations, G5 exhibited the highest cumulative release. At 16 and 24 h, G5 showed significantly higher release than that of most groups (*p* < 0.01).

**Figure 4:**
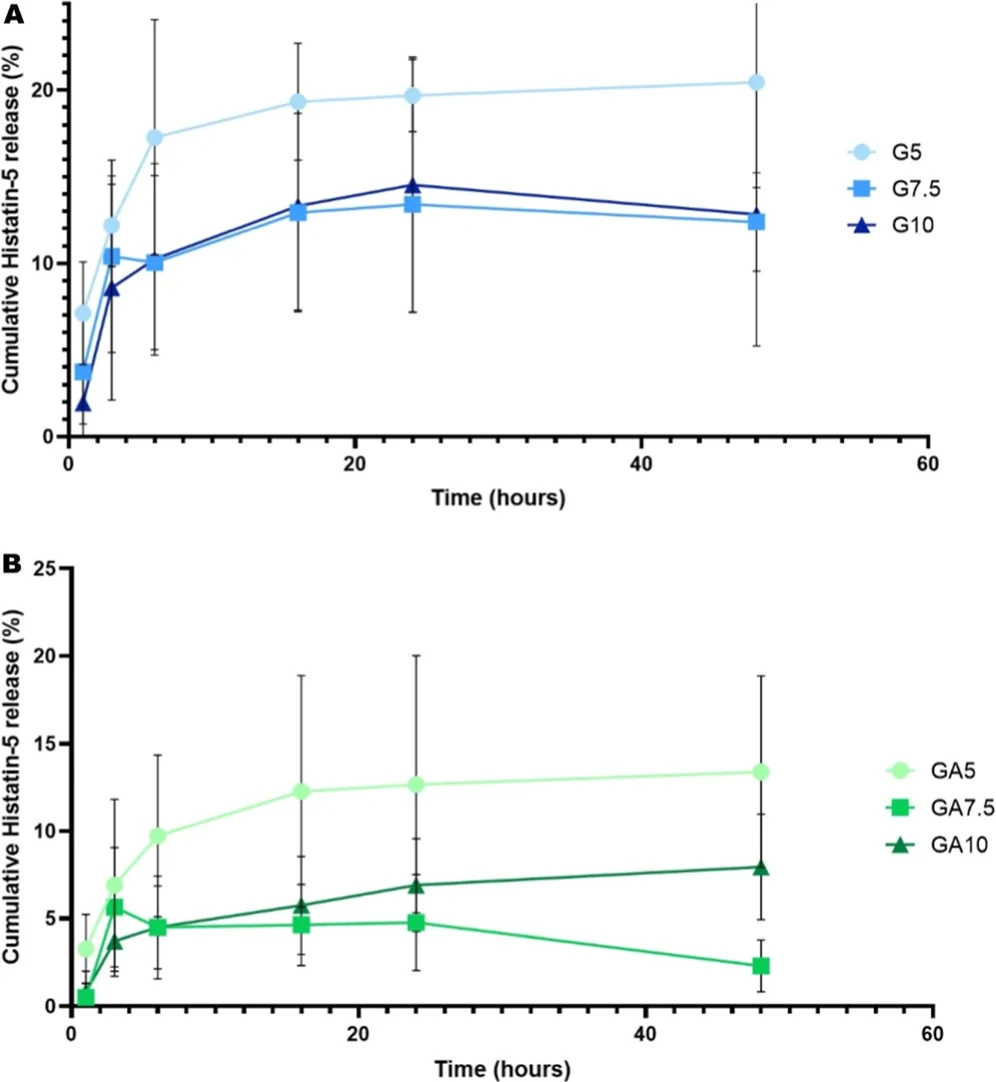
Cumulative release (%) of Histatin-5-FAM from GelMA-based hydrogel formulations over 48 h in PBS at 37 °C. (A) GelMA hydrogels and (B) GelMA-alginate hydrogels. Formulations included GelMA at 5%, 7.5%, and 10% (w/v). Peptide release was quantified at predetermined time points (0, 1, 3, 6, 16, 24, and 48 h) and expressed relative to the initial peptide loading. Data are presented as mean ± standard deviation (*n* = 9).

At 48 h, G5 exhibited a release profile comparable to that of GA5, while remaining significantly higher than that of the other formulations (*p* < 0.006). The lowest release was observed for GA7.5 and GA10, whereas G7.5, G10, and GA5 displayed intermediate release levels. This behavior suggests that lower GelMA concentrations favor faster diffusion of the peptide, whereas the incorporation of alginate likely restricts molecular mobility within the hydrogel network.

### 3.5 Cytocompatibility of GelMA Hydrogels with Human Gingival Fibroblasts (HGFs)

The cytocompatibility of the hydrogels was assessed by evaluating the metabolic activity of human gingival fibroblasts (HGFs) using the Alamar Blue assay at predetermined time points (4, 6, and 24 h). All formulations supported HGF viability and proliferation during the experimental period (Figure 5).

**Figure 5:**
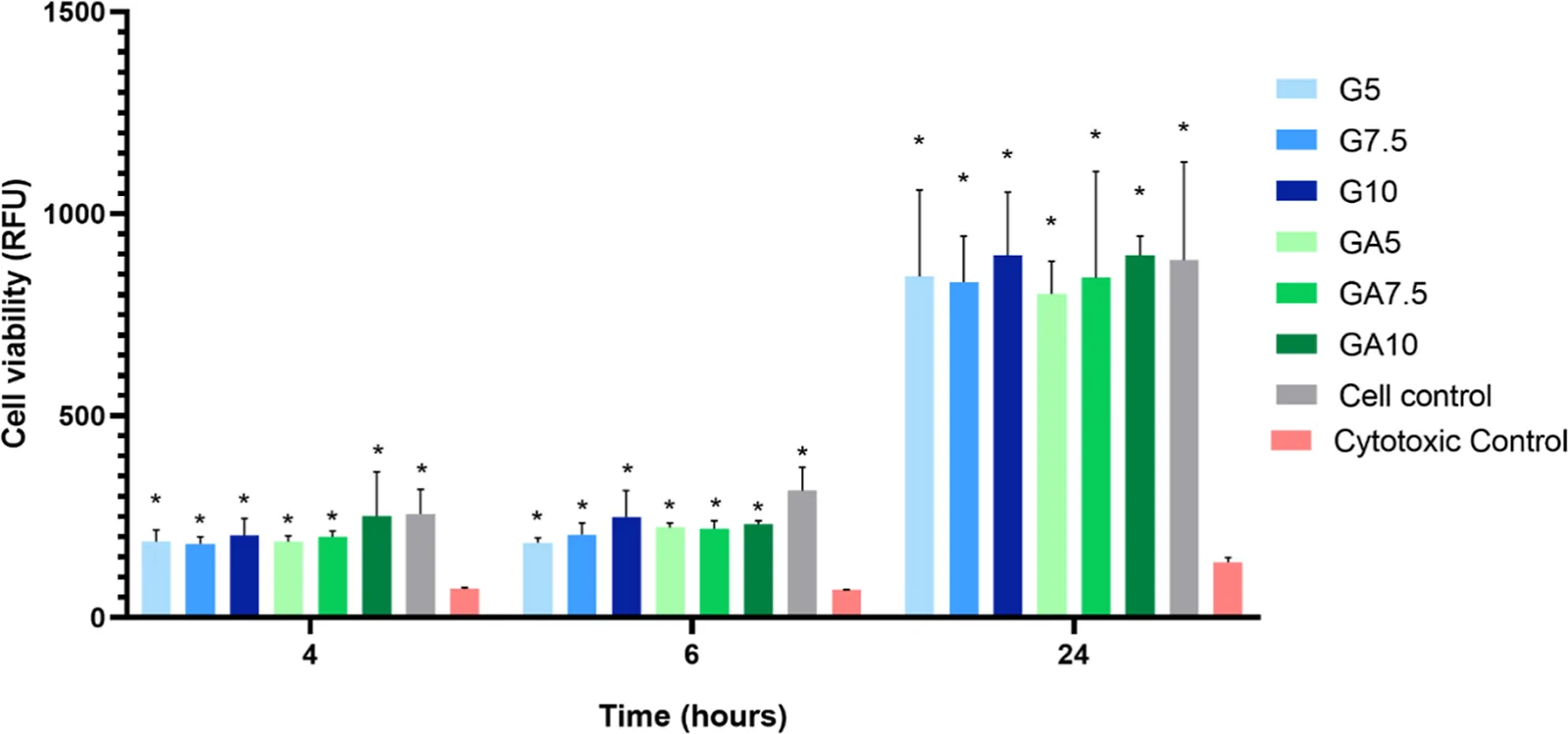
Cell viability of human gingival fibroblasts cultured with GelMA and GelMA-alginate hydrogel formulations evaluated by Alamar Blue assay after 4, 6, and 24 h. Formulations included GelMA at 5%, 7.5%, and 10% (w/v). Data are presented as mean ± standard deviation (*n* = 9). Statistical analysis was performed using two-way ANOVA followed by Tukey’s post hoc test (*p* < 0.05). Asterisks (*) indicate statistically significant differences compared to the cytotoxic control, while different letters indicate significant differences among experimental groups.

At 24 h, all groups exhibited increased fluorescence intensity, with viability values exceeding 100%, indicating enhanced cellular metabolic activity and proliferation relative to baseline. This increase was statistically significant compared with that at earlier time points. No significant differences were observed among the experimental groups; significant differences were detected only in comparison with the cytotoxicity control group.

### 3.6 Antimicrobial Activity of AMP-Loaded GelMA Hydrogels

#### 3.6.1 Colony-Forming Unit (CFU/mL) Analysis of *C. albicans* Biofilms Treated with AMP-Loaded GelMA Hydrogels

Three-way ANOVA revealed a significant interaction among GelMA concentration, AMP type, and alginate incorporation (*p* = 0.002), indicating that alginate modulated AMP efficacy in a formulation-dependent manner.

As shown in Figure 6A, GelMA formulations loaded with INLK and GK-17 exhibited the greatest reduction in *C. albicans* biofilm viability. In the 5% GelMA concentration, G5 loaded with INLK showed a significant reduction compared to G5, G5 + Hst-5, G5 + GK-17, GA5, GA5 + Hst-5, and GA5 + INLK (*p* < 0.03), corresponding to an approximate 1.6-log reduction relative to the GA5 control (without AMP).

**Figure 6:**
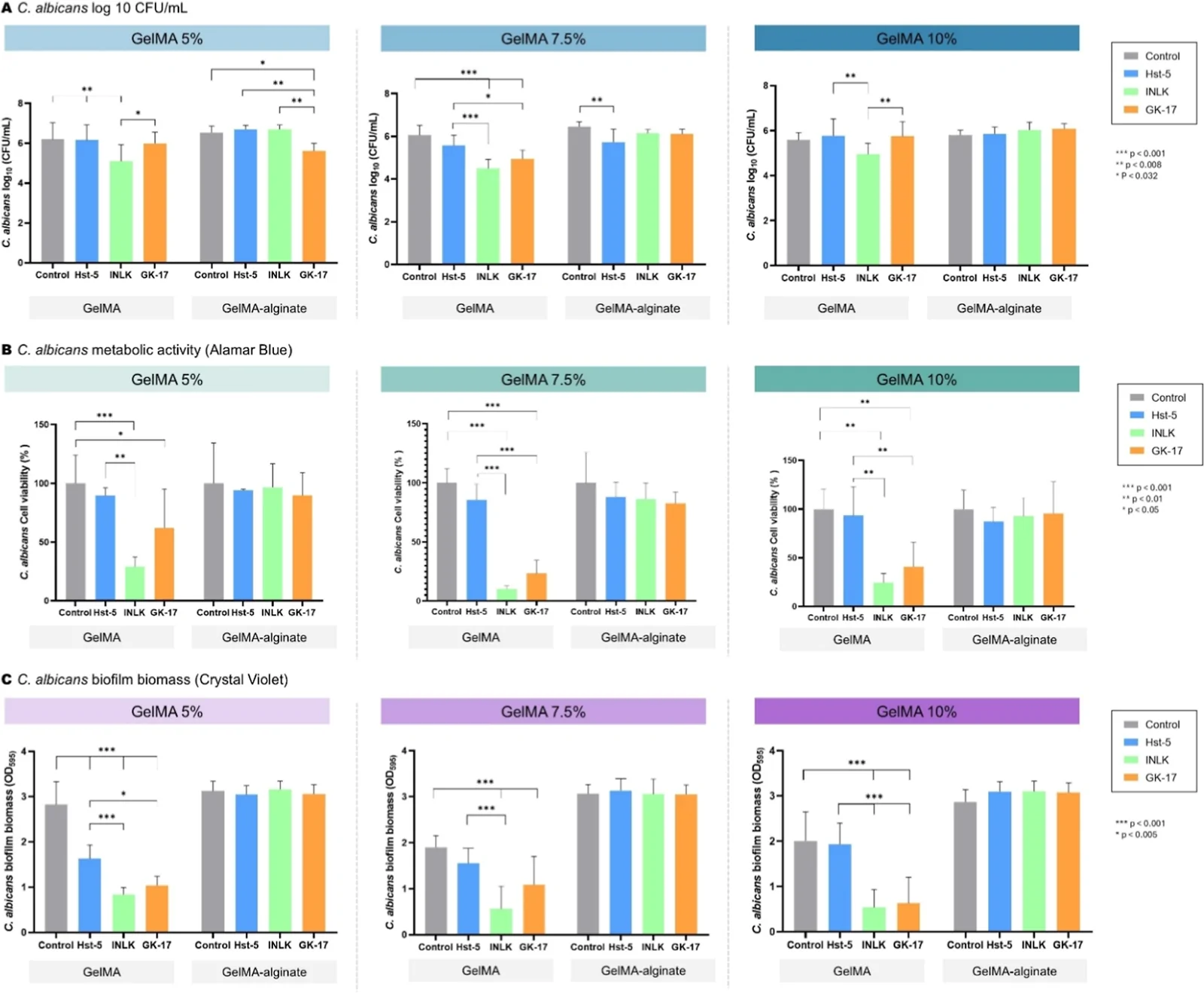
Antifungal activity of GelMA and GelMA–alginate hydrogels loaded with antimicrobial peptides against *Candida albicans* biofilms. (A) Log_10_ CFU/mL after 16 h of treatment. (B) *C. albicans* cell viability (%) assessed by Alamar Blue assay. (C) Biofilm biomass quantified by crystal violet staining (OD_595_). Hydrogels were formulated at 5%, 7.5%, and 10% (w/v), and loaded with Histatin 5 (Hst-5), INLK, or GK-17. Peptide-free hydrogels served as controls. Data are presented as mean ± standard deviation (*n* = 9). Asterisks indicate statistically significant differences between groups.

At a G7.5 concentration loaded with INLK, CFU counts were significantly reduced compared to most groups (*p* < 0.0001); however, no significant difference was observed compared to G7.5 loaded with GK-17 (*p* = 0.28), with all three formulations resulting in approximately 2-log reductions relative to the GA7.5 control.

Similarly, in the 10% GelMA concentration, G10 loaded with INLK exhibited significantly lower CFU counts than the G10 groups, with reductions ranging from 0.9 to 1.2 log units. These findings suggest that the peptide type plays a more critical role than alginate incorporation in determining antifungal efficacy at intermediate GelMA concentrations.

Three-way ANOVA revealed a significant interaction among GelMA concentration, AMP type, and alginate incorporation (*p* = 0.002), indicating that the presence of alginate modulated AMP efficacy in a formulation-dependent manner.

#### 3.6.2 Metabolic Activity of *C. albicans* Cells after Treatment with AMP-Loaded GelMA Hydrogels

A significant interaction was observed among GelMA concentration, AMP type, and alginate incorporation (2%; *p* = 0.001). Overall, GelMA formulations exhibited greater efficacy in reducing the metabolic activity of *C. albicans* than GelMA–alginate formulations. Among the tested AMPs, INLK-loaded GelMA hydrogels showed the greatest reduction in metabolic viability, with values of 28.9%, 10.3%, and 24.6% for G5, G7.5, and G10, respectively. Similarly, GK-17 significantly reduced metabolic viability in the G7.5 and G10 formulations, reaching 23.5% and 40.9%, respectively, whereas G5 achieved a viability of 61.9% (*p* < 0.05). In contrast, GelMA–alginate formulations exhibited considerably higher metabolic viability, ranging from 82.9% to 96.6%, regardless of the incorporated AMP, indicating a markedly lower antimicrobial effect (Figure 6B). These findings suggest that peptide activity is strongly influenced by hydrogel composition, with GelMA matrices favoring substantially greater antimicrobial efficacy than GelMA–alginate formulations.

#### 3.6.3 Quantification of *C. albicans* Total Biomass after Treatment with AMP-Loaded GelMA Hydrogels

Significant interactions were observed between GelMA concentration and alginate incorporation (*p* = 0.0001) and between alginate incorporation and AMP type (*p* = 0.00001), indicating that biofilm biomass was strongly influenced by both hydrogel composition and peptide loading. Overall, GelMA–alginate formulations exhibited consistently higher absorbance values than GelMA-only hydrogels, suggesting greater *C. albicans* biofilm biomass formation in the presence of alginate.

Among the GelMA-only formulations, AMP incorporation significantly reduced biofilm biomass compared to of that the unloaded controls (Figure 6C). In the G5 and G7.5 groups, all the tested peptides promoted substantial biomass reduction, with INLK exhibiting the lowest absorbance values, followed by GK-17. Histatin-5 also reduced biomass formation, but to a lesser extent. In the G10 formulations, AMP-loaded hydrogels similarly demonstrated reduced biomass compared to the control group, with INLK and GK-17 performing a greater reduction in biofilm biomass than Hist-5.

In contrast, alginate-containing formulations showed limited biomass reduction, regardless of the incorporated peptide, with absorbance values comparable to their respective controls. This behavior suggests that alginate incorporation may have restricted AMP diffusion and reduced peptide availability at the biofilm interface, thereby compromising antibiofilm activity despite the presence of antimicrobial peptides.

#### 3.6.4 Evaluation of *C. albicans* Viability by Confocal Laser Scanning Microscopy (CLSM)

Confocal laser scanning microscopy (CLSM) images (Figures 7 and 8) illustrate the viability of *C. albicans* biofilms following treatment with AMP-loaded GelMA formulations. In control groups and Histatin 5-treated GelMA hydrogels without alginate, predominantly green fluorescence was observed, indicating a high proportion of viable cells. In contrast, hydrogels loaded with INLK and GK-17 showed a predominance of red fluorescence, indicating extensive loss of cell viability. These observations corroborate the CFU counts, metabolic activity (Alamar Blue), and biomass (crystal violet) assays.

**Figure 7:**
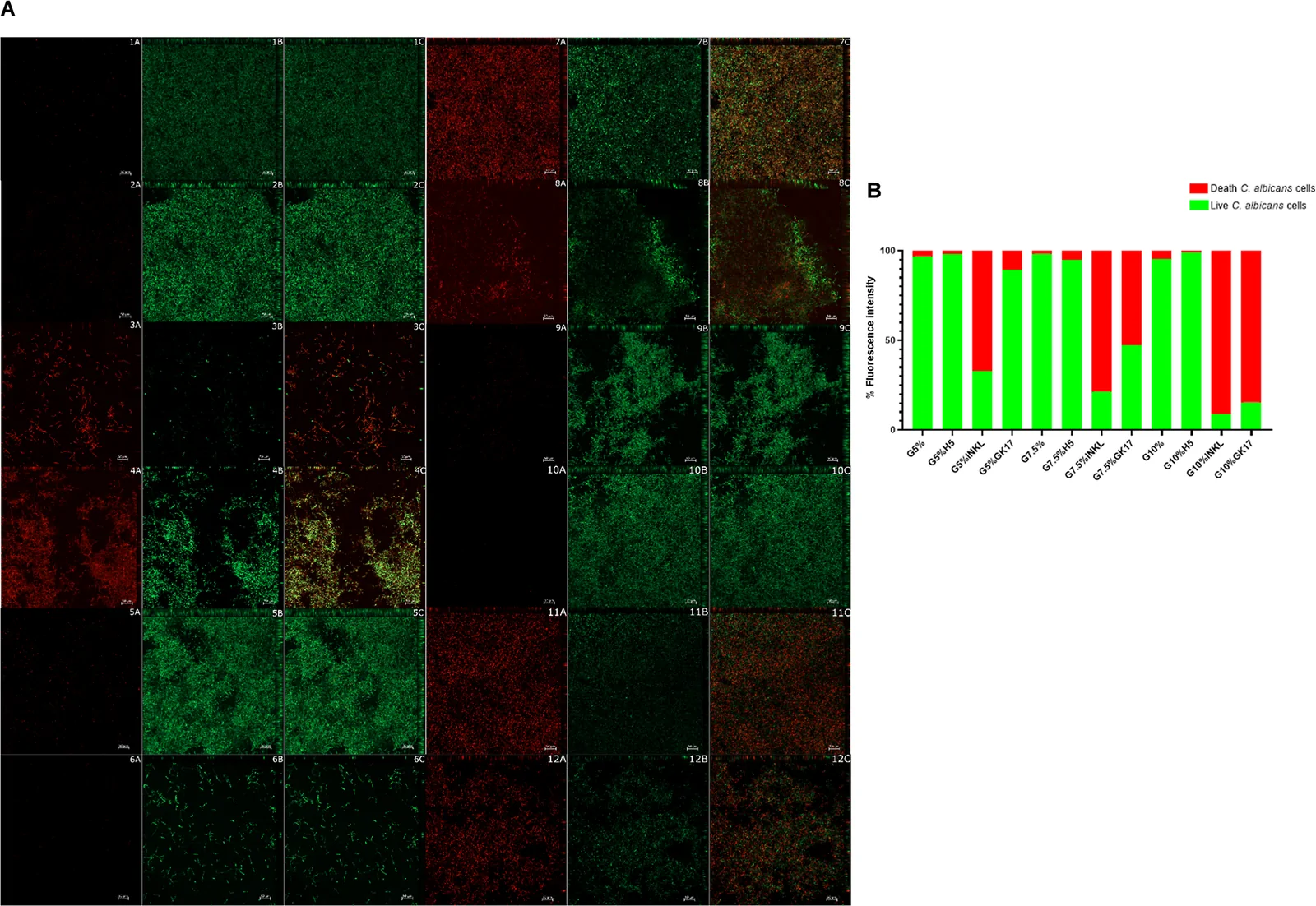
(A) Viability of *Candida albicans* biofilms analyzed by confocal laser scanning microscopy (CLSM) after 16 h of treatment with GelMA hydrogel formulations (5%, 7.5%, and 10% w/v) loaded with antimicrobial peptides (Histatin 5, INLK, and GK-17). Peptide-free hydrogels served as controls. Biofilms were stained with SYTO-9 (live cells, green) and propidium iodide (dead cells, red). Representative images are shown as a- dead cells (PI), b- live cells (SYTO-9), and c- merged images. Samples are organized as follows:^1–4^ GelMA 5%,^5–8^ GelMA 7.5%, and^9–12^ GelMA 10%, corresponding to control, Histatin 5, INLK, and GK-17, respectively. (B) Quantification of fluorescence intensity, expressed as the percentage of live (green) and dead (red) cells within the biofilms.

**Figure 8:**
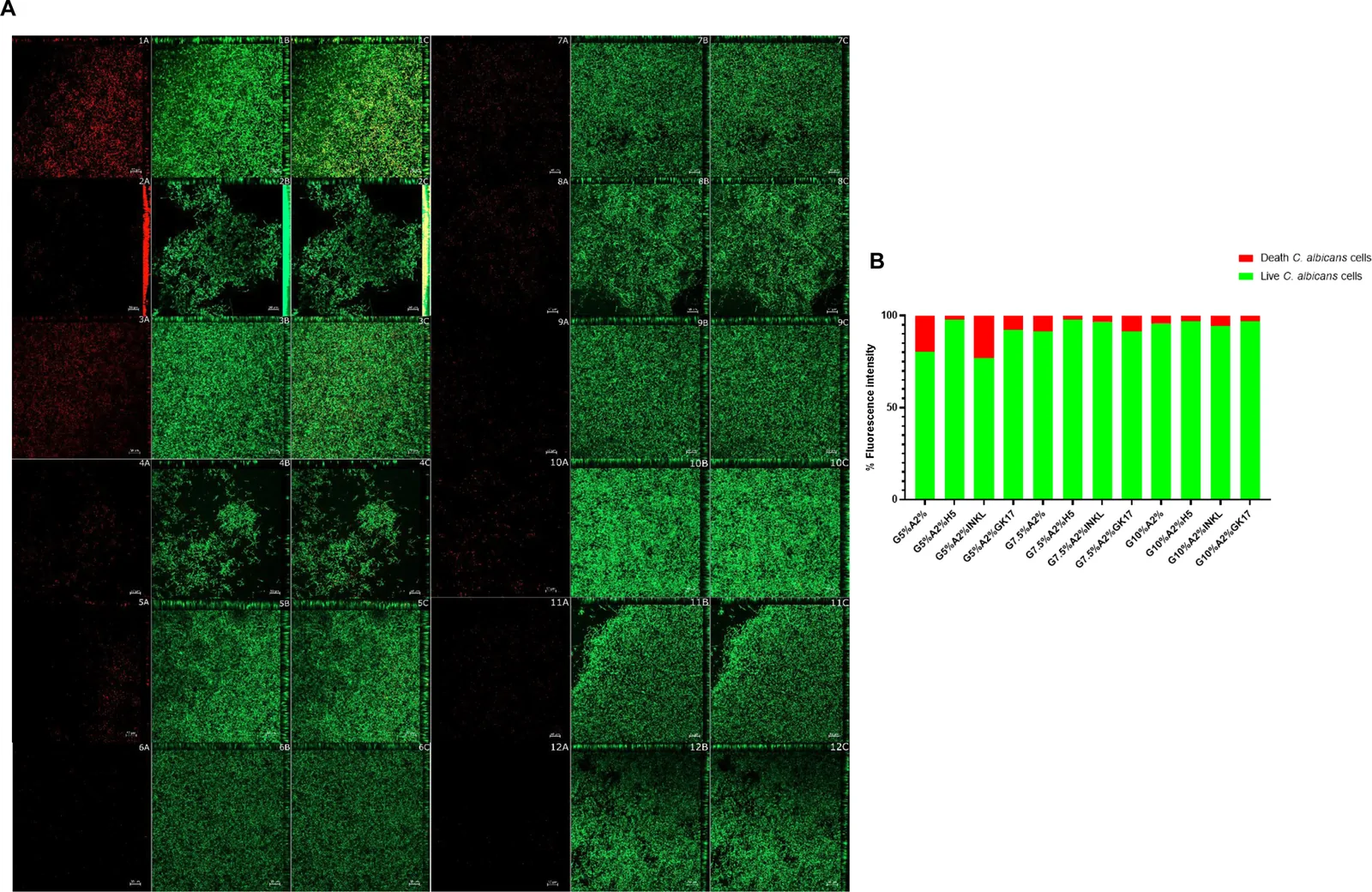
(A) Viability of *Candida albicans* biofilms analyzed by confocal laser scanning microscopy (CLSM) after 16 h of treatment with GelMA-alginate hydrogel formulations (5%, 7.5%, and 10% w/v) loaded with antimicrobial peptides (Histatin 5, INLK, and GK-17). Peptide-free hydrogels served as controls. Biofilms were stained with SYTO-9 (live cells, green) and propidium iodide (dead cells, red). Representative images are shown as a- dead cells (PI), b- live cells (SYTO-9), and c- merged images. Samples are organized as follows:^1–4^ GelMA 5%,^5–8^ GelMA 7.5%, and^9–12^ GelMA 10%, corresponding to control, Histatin 5, INLK, and GK-17, respectively. (B) Quantification of fluorescence intensity, expressed as the percentage of live (green) and dead (red) cells within the biofilms.

## 4 Discussion

The null hypothesis was rejected because significant differences were observed among GelMA formulations, AMP type, and alginate incorporation. Overall, GelMA hydrogels exhibited favorable physicochemical and biological properties, including adequate moldability, tunable mechanical behavior, sustained peptide release, and cytocompatibility with human gingival fibroblasts. Importantly, GelMA formulations without alginate demonstrated superior efficacy in reducing *C. albicans* biofilms, while INLK exhibited the strongest antimicrobial activity among the tested peptides. These findings indicate that both the hydrogel composition and peptide type play critical roles in modulating antimicrobial performance. Collectively, these results underscore the potential of GelMA-based hydrogels as tunable delivery platforms for antimicrobial peptides, offering controlled release, cytocompatibility, and effective biofilm disruption. Together, these findings support the advancement of hydrogel-based strategies for the localized treatment of biofilm-associated infections.

The physicochemical properties of GelMA are fundamental to understanding the behavior of this system. Previous studies^20,30,31^ have demonstrated that GelMA concentration, degree of methacrylation, and cross-linking density are key parameters governing the mechanical properties and network architecture of hydrogels. In agreement with these reports, GelMA solutions exhibited a viscous prepolymer state prior to light exposure (385–515 nm), transitioning into a solid hydrogel upon photo-cross-linking.^32^

SEM analysis confirmed that GelMA hydrogels presented a porous structure that was not markedly altered by the incorporation of alginate (2%). However, increasing GelMA concentration resulted in reduced pore size, consistent with previous findings showing an inverse relationship between pore size and polymer concentration, as well as the influence of the degree of methacrylation on network organization,^19^ a crucial metric for photo-cross-linkable materials like GelMA.

Viscosity is a critical parameter that influences the handling, injectability, and structural stability of hydrogels. The viscosity of GelMA and GelMA–alginate formulations increases proportionally with GelMA concentration.^30,33,34^ In particular, higher GelMA concentrations combined with alginate have been reported to exhibit increased viscosity compared to lower concentrations of GelMA.^34^ Supporting these findings, the GelMA–alginate formulations in the present study exhibited higher viscosity than GelMA alone. Despite this increase, all formulations exhibited shear-thinning behavior and maintained rheological profiles compatible with injectability. Additionally, the observed thermal stability suggests that these hydrogels can maintain their structural integrity under physiological conditions, supporting their potential for biomedical applications. This balance between viscosity and shear-thinning behavior is particularly important for ensuring ease of application and retention at the target site.

The compressive mechanical properties of hydrogels play a key role in bioadhesion and cell–matrix interactions.^35^ The compressive modulus of GelMA-based hydrogels can be tuned by varying parameters such as the degree of methacrylation, photoinitiator concentration, and cross-linking density.^20,30,31,34,35^ Although GelMA hydrogels often exhibit limited mechanical robustness for load-bearing or long-term applications, the incorporation of alginate significantly enhances their stiffness. This reinforcing effect is further amplified at higher GelMA concentrations.^36^ In line with these reports, the GA7.5 and GA10 formulations in the present study exhibited compressive modulus values more than 10-fold higher than those of the G5 and G7.5 groups, indicating a substantial improvement in mechanical integrity. This enhancement in mechanical properties may contribute to improved structural stability and retention at the application site.

The degradation and swelling behaviors are key physicochemical properties that influence the performance of GelMA hydrogels in bioadhesive applications. These properties are governed by several factors, including GelMA and photoinitiator concentrations, pore structure formed during cross-linking, degree of methacrylation, and the overall hydrophilicity of the hydrogel matrix.^20,31,35^ Together, these parameters regulate mass transport and diffusion within the hydrogel network, which impacts the release of incorporated molecules. Excessive swelling may compromise structural integrity and reduce adhesion performance.^35^

The incorporation of alginate into the hydrogel composition significantly influenced the degradation and swelling behavior. The GA5 formulation exhibited the greatest mass loss and the lowest swelling ratio after 48 h among all groups. This behavior may be attributed to the combination of low GelMA concentration and alginate incorporation, resulting in a less densely cross-linked network that is more susceptible to hydrolytic degradation.^31^ In contrast, hydrogels with higher GelMA concentrations exhibited slower degradation rates, likely because of their more compact network structure, characterized by smaller pore sizes and increased cross-linking density.^37^

These structural characteristics have direct implications for drug release behavior. Analysis of the release kinetics of GelMA hydrogels loaded with Hst-5–FAM showed that more porous and faster-degrading matrices, such as G5, exhibited higher peptide release. As the GelMA concentration increased, the release rate decreased, likely due to the formation of a denser and more tightly cross-linked network that restricts peptide diffusion.^38^ The incorporation of alginate, particularly in formulations with higher GelMA concentrations, further reduced Hst-5–FAM release, suggesting that increased network density promotes sustained release through limited diffusion and possible peptide entrapment within the hydrogel matrix.^39^ This controlled release profile may support the maintenance of effective local peptide concentrations, enhancing the therapeutic potential of GelMA-based bioadhesive systems.

GelMA hydrogels are widely recognized for their excellent cytocompatibility, which can be modulated by parameters such as the degree of methacrylation.^38^ Previous studies have shown that GelMA supports the formation of complex cellular structures and tissue-like organization, highlighting its potential for applications in tissue engineering and wound healing.^40^ The network architecture also plays a key role in cellular responses; increasing GelMA concentration has been associated with the formation of denser networks, which may limit cell spreading and proliferation.^37^ Shie et al. reported that human dermal fibroblasts maintained viability above 90% across all groups, with enhanced proliferation observed at lower GelMA concentrations.^37^ In agreement with these findings, the present study demonstrated that human gingival fibroblasts cultured on GelMA hydrogels exhibited metabolic activity exceeding 100% after 24 h. Rather than reflecting mere cytocompatibility, this result indicates a stimulatory effect on fibroblast metabolic activity, consistent with enhanced cell proliferation, possibly driven by the supportive nature of the GelMA matrix itself. Similar results have been reported for GelMA-based systems incorporating bioactive components, such as CeO_2_ nanoparticles, which also supported HGF viability.^19^

Regarding antimicrobial performance, unloaded GelMA and GelMA–alginate hydrogels exhibited no intrinsic antifungal activity against *C. albicans*, consistent with previous reports demonstrating the limited antimicrobial effect of GelMA matrices in the absence of bioactive agents.^32^ Interestingly, alginate-containing formulations also failed to reduce biofilm formation, which may be associated not only with their elevated water-retention capacity but also with alterations in hydrogel network architecture that potentially favor fungal persistence. The highly hydrated microenvironment generated by alginate-rich matrices may facilitate nutrient diffusion and maintain conditions favorable for *C. albicans* survival and biofilm stability.^41^

In contrast, GelMA hydrogels without alginate loaded with INLK exhibited the highest antifungal efficacy, achieving approximately a 2-log reduction in CFU/mL and reducing *C. albicans* metabolic viability to 10.3–28.9% of the untreated control. This was followed by G7.5 and G10 formulations loaded with GK-17, which reduced metabolic viability to 23.5% and 40.9%, respectively. These findings were consistently corroborated by biomass quantification and CLSM analyses, which demonstrated a predominance of nonviable cells in INLK- and GK-17-loaded GelMA formulations, whereas alginate-containing hydrogels maintained high metabolic viability (82.9–96.6% of the untreated control) and predominantly viable biofilms.

The enhanced antifungal performance of INLK and GK-17 may be associated with both peptide-specific mechanisms and hydrogel-mediated modulation of peptide bioavailability. GK-17, a derivative of LL-37, has been reported to exhibit potent antifungal activity combined with increased proteolytic stability,^16^ while INLK, derived from wasp venom, demonstrates strong membrane–disruptive activity against *Candida* species.^42^ Moreover, the superior performance of GelMA-only formulations suggests that the hydrogel composition critically influences AMP diffusion and therapeutic availability. We hypothesize that the incorporation of alginate may have increased network density and diffusional tortuosity, while simultaneously altering the ionic microenvironment within the hydrogel matrix. Due to its highly anionic nature, alginate contains negatively charged carboxyl groups that are presumably capable of establishing electrostatic interactions with the cationic AMPs used in this study,^43^ although this interaction was not directly assessed and remains to be experimentally confirmed. Such ionic interactions may have promoted peptide sequestration within the hydrogel network, potentially reducing peptide mobility, limiting diffusion toward the biofilm interface, and ultimately decreasing AMP bioavailability.

Conventional antifungal therapies for oral candidiasis, including nystatin and amphotericin B, remain effective but face increasing limitations. Antifungal resistance has become widespread, driven by prolonged therapy, frequent prophylaxis, subtherapeutic dosing, and biofilm-mediated drug sequestration.^44^ In addition, nystatin regimens typically require administration up to four times daily for several weeks, with no universally accepted dosing protocol, which may compromise patient adherence.^45^ Chlorhexidine, widely used as an oral antiseptic, has similarly been shown to induce the formation of drug-tolerant *C. albicans* persister cells within biofilms, which can resuscitate after treatment discontinuation and contribute to recurrent infection.^46^ In contrast, antimicrobial peptides act primarily through rapid membrane disruption or intracellular targeting, which may reduce the likelihood of resistance selection compared to conventional agents. When incorporated into GelMA hydrogels, these peptides can further benefit from sustained local release and prolonged retention at the application site, potentially enhancing therapeutic efficacy while minimizing systemic exposure and dosing frequency associated with conventional formulations.

Hydrogel-based drug delivery systems have emerged as promising platforms for the localized treatment of oral infections, improving drug retention and enabling controlled release. Most previously reported hydrogel systems for oral candidiasis have focused on the delivery of essential oils or other natural antifungal compounds rather than peptides, such as clove and lemongrass essential oil-loaded hydrogels^47^ and citral-based hydrogels.^48^ Similar antimicrobial outcomes have been reported for GelMA-based AMP delivery systems, although these primarily target bacterial infections rather than fungal pathogens.^22–24,32,49^ In contrast, the present study explored GelMA hydrogels as carriers for antimicrobial peptides specifically against *C. albicans*, combining the tunable physicochemical properties of GelMA with the potent antimicrobial activity of host-derived and synthetic peptides. This strategy not only provides sustained peptide delivery but also offers the possibility of modulating hydrogel properties according to the intended clinical application, thereby supporting the development of multifunctional biomaterials for oral fungal infection management.

Several limitations of this study must be acknowledged. First, all experiments were conducted under in vitro conditions, which do not fully recapitulate the complexity of the oral environment. In particular, the absence of saliva represents a gap, as salivary components, including proteins, enzymes, mucins, and electrolytes, are known to influence hydrogel swelling, degradation, and bioadhesion, as well as AMP stability and bioavailability. Salivary proteases may accelerate peptide degradation, while salivary proteins may compete with AMPs for hydrogel binding sites or interact electrostatically with cationic peptides, potentially reducing their effective concentration at the biofilm interface. Second, the biofilm model employed here, while well-established, does not reflect the structural heterogeneity, metabolic diversity, or polymicrobial composition of clinical oral biofilms, which typically involve complex interactions between fungal and bacterial species embedded within a mature extracellular matrix. Third, no in vivo validation was performed, and the long-term bioadhesive performance, tissue compatibility, and therapeutic efficacy of these hydrogels within a living oral mucosa remain to be established. Fourth, release kinetics were characterized only for Hst-5–FAM, and it was assumed that GK-17 and INLK would exhibit similar diffusion behavior based on their shared cationic character. However, the three peptides differ in net charge, molecular size, and hydrophobicity, all of which can independently influence diffusion through and electrostatic interaction with the GelMA-alginate network; consequently, GK-17 and INLK may display distinct release profiles that were not directly captured in this study, and this assumption should be confirmed through peptide-specific release characterization in future work. Fifth, the present experimental design did not include a clinically relevant positive control, such as chlorhexidine or a conventional antifungal agent (e.g., nystatin); benchmarking against such agents in future studies would provide a valuable reference for interpreting the translational potential of the antibiofilm efficacy observed here. Despite these limitations, the present findings demonstrate that GelMA-based hydrogels loaded with AMPs can effectively reduce *C. albicans* biofilms. These results provide a foundation for the development of bioadhesive delivery systems capable of sustained AMP release at the oral mucosa. Such approaches may offer a localized therapeutic strategy to control *C. albicans* biofilm-associated infections and support tissue healing, while potentially reducing the risk of antimicrobial resistance.

In conclusion, GelMA-based hydrogels exhibit favorable physicochemical and biological properties, including cytocompatibility, tunable viscoelastic behavior, structural stability, and sustained AMP release. Hydrogel composition was shown to critically modulate peptide bioavailability and antifungal performance against *C. albicans* biofilms, with GelMA formulations without alginate exhibiting superior antimicrobial efficacy, particularly when loaded with INLK and GK-17 peptides. This suggests that the increased network density and ionic interactions associated with alginate incorporation may have restricted peptide diffusion and reduced AMP availability at the biofilm interface. These findings underscore the importance of rationally tuning the hydrogel network architecture to balance mechanical reinforcement and therapeutic diffusivity, supporting the potential of GelMA-based systems as promising biomaterial platforms for the localized and controlled delivery of AMPs in the management of *C. albicans*-associated oral biofilm infections. Nevertheless, further in vivo and translational studies are necessary to validate their long-term performance, bioadhesion, stability in the oral environment, and clinical applicability.

## Notes

This study was financed in part by the Coordenação de Aperfeiçoamento de Pessoal de Nível Superior—Brasil (CAPES)—Finance Code 001.

## References

[1] Vanhoecke B., De Ryck T., Stringer A., Van de Wiele T., Keefe D. (2015). Microbiota and their role in the pathogenesis of oral mucositis. Oral Diseases.

[2] Hwang G. (2022). In it together:
Candida-bacterial oral biofilms and therapeutic strategies. Environ. Microbiol. Rep..

[3] Pereira C. A., Toledo B. C., Santos C. T., Pereira Costa A. C., Back-Brito G. N., Kaminagakura E., Jorge A. O. (2013). Opportunistic microorganisms
in individuals with lesions of denture stomatitis. Diagn. Microbiol. Infect. Dis..

[4] Arzmi M. H., Dashper S., McCullough M. (2019). Polymicrobial
interactions of *Candida albicans* and its role in
oral carcinogenesis. J. Oral Pathol. Med..

[5] Guo J., Zhang M., Qiao D., Shen H., Wang L., Wang D., Li L., Liu Y., Lu H., Wang C. (2021). Prevalence and antifungal
susceptibility of *Candida parapsilosis* species complex
in Eastern China: A
15-year retrospective study by ECIFIG. Front.
Microbiol..

[6] Jiao Y., Tay F. R., Niu L. N., Chen J. H. (2019). Advancing antimicrobial
strategies for managing oral biofilm infections. Int. J. Oral Sci..

[7] Antimicrobial
Resistance Collaborators (2022). Global burden of bacterial antimicrobial resistance in 2019: A systematic
analysis. Lancet.

[8] Alvarez L., Kumaran K. S., Nitha B., Sivasubramani K. (2025). Evaluation
of biofilm formation and antimicrobial susceptibility (drug resistance)
of *Candida albicans* isolates. Braz. J. Microbiol..

[9] Bucataru C., Ciobanasu C. (2024). Antimicrobial peptides: Opportunities
and challenges
in overcoming resistance. Microbiol. Res..

[10] Swidergall M., Ernst J. F. (2014). Interplay between *Candida
albicans* and the antimicrobial peptide armory. Eukaryotic
Cell.

[11] Mesa A., Orrego A., Branch-Bedoya J. W., Mera-Banguero C., Orduz S. (2025). Antimicrobial peptides design using
deep learning and rational modifications:
Activity in bacteria, Candida albicans, and cancer cells. Curr. Microbiol..

[12] Moffa E. B., Mussi M. C., Xiao Y., Garrido S. S., Machado M. A., Giampaolo E. T., Siqueira W. L. (2015). Histatin 5 inhibits adhesion of *C. albicans* to reconstructed human oral epithelium. Front.
Microbiol..

[13] Fan L., Sun J., Zhou M., Zhou J., Lao X., Zheng H., Xu H. D. R. A. M. P. (2016). A comprehensive data repository of
antimicrobial peptides. Sci. Rep..

[14] Zolin G. V. S., Fonseca F. H., Zambom C. R., Garrido S. S. (2021). Histatin 5 metallopeptides
and their potential against *Candida albicans* pathogenicity
and drug resistance. Biomolecules.

[15] Cesa-Luna C., Muñoz-Rojas J., Saab-Rincon G., Baez A., Morales-García Y. E., Juárez-González V. R., Quintero-Hernández V. (2019). Structural
characterization of scorpion peptides and their bactericidal activity
against clinical isolates of multidrug-resistant bacteria. PLoS One.

[16] Kalimuthu S., Pudipeddi A., Braś G., Tanner J. A., Rapala-Kozik M., Leung Y. Y., Neelakantan P. (2023). A heptadeca
amino acid peptide subunit
of cathelicidin LL-37 has previously unreported antifungal activity. APMIS.

[17] Kalimuthu K., Srinivasan N. R., Govindarajan G. (2023). Antibiotic-peptide conjugation against
multi-drug resistant pathogens: A comprehensive review for therapeutics
and drug delivery strategies. Int. J. Pept.
Res. Ther..

[18] Ansari S., Pouraghaei Sevari S., Chen C., Sarrion P., Moshaverinia A. (2021). RGD-modified
alginate-GelMA hydrogel sheet containing gingival mesenchymal stem
cells: A unique platform for wound healing and soft tissue regeneration. ACS Biomater. Sci. Eng..

[19] Yang F., He Y., Wang F., Zhao X., Hsu Y., Yan F., Zhou X., Hu W., Xia D., Liu Y. (2025). Antibacterial
and antioxidant GelMA/CeO2 hydrogel promotes oral mucosal healing. J. Dent. Res..

[20] Yue K., Trujillo-de Santiago G., Alvarez M. M., Tamayol A., Annabi N., Khademhosseini A. (2015). Synthesis, properties, and biomedical
applications
of gelatin methacryloyl (GelMA) hydrogels. Biomaterials.

[21] Zhu W., Dong Y., Xu P., Pan Q., Jia K., Jin P., Zhou M., Xu Y., Guo R., Cheng B. (2022). A composite
hydrogel containing resveratrol-laden nanoparticles and platelet-derived
extracellular vesicles promotes wound healing in diabetic mice. Acta Biomater..

[22] Liang C., Wang H., Lin Z., Zhang C., Liu G., Hu Y. (2023). Augmented wound healing
potential of photosensitive GelMA hydrogel
incorporating antimicrobial peptides and MXene nanoparticles. Front. Bioeng. Biotechnol..

[23] Ch S., Mishra P., Padaga S. G., Ghosh B., Roy S., Biswas S. (2024). 3D-printed inherently
antibacterial contact lens-like
patches carrying antimicrobial peptide payload for treating bacterial
keratitis. Macromol. Biosci..

[24] Pulat G., Çelebi N. N., Bilgiç E. (2025). The effect
of immobilization methods
of P9–4 antimicrobial peptide onto gelatin methacrylate on
multidrug-resistant bacteria: A comparative study. Macromol. Biosci..

[25] Krishnamoorthy S., Zhang Z., Xu C. (2019). Biofabrication
of three-dimensional
cellular structures based on gelatin methacrylate–alginate
interpenetrating network hydrogel. J. Biomater.
Appl..

[26] Ma C., Kim Y.-K., Lee M.-H., Jang Y.-S. (2023). Development of gelatin
methacryloyl/sodium alginate interpenetrating polymer network hydrogels
for bone regeneration by activating the Wnt/β-catenin signaling
pathway via lithium release. Int. J. Mol. Sci..

[27] Hafezi M., Khorasani S. N., Khalili S., Neisiany R. E. (2023). Self-healing
interpenetrating
network hydrogel based on GelMA/alginate/nano-clay. Int. J. Biol. Macromol..

[28] Marfoglia A., Tibourtine F., Pilloux L., Cazalbou S. (2023). Tunable double-network
GelMA/alginate hydrogels for platelet lysate-derived protein delivery. Bioengineering.

[29] da
Silva I. S. P., Bordini E. A. F., Bronze-Uhle E. S., de Stuani V., Costa M. C., de Carvalho L. A. M., Cassiano F. B., de Azevedo Silva L. J., Borges A. F. S., Soares D. G. (2024). Photo-crosslinkable
hydrogel incorporated with bone matrix particles for advancements
in dentin tissue engineering. J. Biomed. Mater.
Res., Part A.

[30] Van
Den Bulcke A. I., Bogdanov B., De Rooze N., Schacht E. H., Cornelissen M., Berghmans H. (2000). Structural and rheological properties
of methacrylamide modified gelatin hydrogels. Biomacromolecules.

[31] Leu
Alexa R., Iovu H., Ghitman J., Serafim A., Stavarache C., Marin M. M., Ianchis R. (2021). 3D-printed gelatin
methacryloyl-based scaffolds with potential application in tissue
engineering. Polymers.

[32] Ren D., Zhang Y., Du B., Wang L., Gong M., Zhu W. (2024). An antibacterial, conductive nanocomposite hydrogel coupled with
electrical stimulation for accelerated wound healing. Int. J. Nanomed..

[33] Kurian A. G., Singh R. K., Lee J. H., Kim H. W. (2022). Surface-engineered
hybrid gelatin methacryloyl with nanoceria as reactive oxygen species
responsive matrixes for bone therapeutics. ACS
Appl. Bio Mater..

[34] Wu S. (2024). Alginate/GelMA microparticles via oil-free interface shearing for
untethered magnetic microbots. Biomater. Sci..

[35] Balu R., Arunachalam N., Dutta N. K., Choudhury N. R. (2025). Gelatin
methacrylate-based bioadhesive hybrid hydrogels by photoclick thiol-ene
chemistry. Polymer.

[36] de
Souza J. R., Rahimnejad M., Mendes Soares I. P., Anselmi C., de Oliveira P. H. C., Dos Reis-Prado A. H., Maglaras V., Dal-Fabbro R., Trichês E. S., Bottino M. C. (2025). 3D printing β-TCP-laden GelMA/alginate interpenetrating-polymer-network
biomaterial inks for bone tissue engineering. Bioprinting.

[37] Shie M.-Y., Lee J.-J., Ho C.-C., Yen S.-Y., Ng H. Y., Chen Y.-W. (2020). Effects of gelatin
methacrylate bio-ink concentration
on mechano-physical properties and human dermal fibroblast behavior. Polymers.

[38] Piao Y., You H., Xu T., Bei H. P., Piwko I. Z., Kwan Y. Y., Zhao X. (2021). Biomedical
applications of gelatin methacryloyl hydrogels. Eng. Regener..

[39] Vigata M., Meinert C., Pahoff S., Bock N., Hutmacher D. W. (2020). Gelatin
methacryloyl hydrogels control the localized delivery of albumin-bound
paclitaxel. Polymers.

[40] Zhao X., Sun X., Yildirimer L., Lang Q., Lin Z. Y. W., Zheng R., Zhang Y., Cui W., Annabi N., Khademhosseini A. (2017). Cell infiltrative
hydrogel fibrous scaffolds for accelerated wound healing. Acta Biomater..

[41] Pham L. H. P., Ly K. L., Colon-Ascanio M., Ou J., Wang H., Lee S. W., Wang Y., Choy J. S., Phillips K. S., Luo X. (2023). Dissolvable alginate hydrogel-based
biofilm microreactors for antibiotic
susceptibility assays. Biofilm.

[42] Luo L., Kamau P. M., Lai R. (2022). Bioactive peptides and proteins from
wasp venoms. Biomolecules.

[43] Wu T., Wang Q., Zhou S., Chen H., Zhang L. (2026). pH-tunable
electro-responsive sodium alginate/chitosan-based Janus polyelectrolyte
hydrogel with reversible bidirectional bending. Int. J. Biol. Macromol..

[44] Tkaczyk M., Kuśka-Kielbratowska A., Fiegler-Rudol J., Niemczyk W., Mertas A., Skaba D., Wiench R. (2025). The Prevalence
and Drug Susceptibility of Candida Species and an Analysis of Risk
Factors for Oral Candidiasis-A Retrospective Study. Antibiotics.

[45] Rai A., Misra S. R., Panda S., Sokolowski G., Mishra L., Das R., Lapinska B. (2022). Nystatin Effectiveness
in Oral Candidiasis Treatment: A Systematic Review & Meta-Analysis
of Clinical Trials. Life.

[46] Lin Y., Cheng L., Zhou X., Liao G., Zhu Z., Ren B. (2026). Chlorhexidine and Polident Induce the Formation of *Candida
albicans* Persistence. Sichuan Da Xue
Xue Bao Yi Xue Ban..

[47] Kola-Mustapha A. T., John F. T., Bello R. H., Adedeji O. J., Ghazali Y. O., Khalid-Salako F. A., Ambrose G. O. (2026). Essential oil-based hydrogels for
oral candidiasis: preliminary formulation development, antifungal
efficacy, and computational analysis via Monte Carlo simulation. Front. Cell. Infect. Microbiol..

[48] Freire J. C. P., Ribeiro A. D., Figueiredo Junior E.
C., da Silva D. T. C., Ribeiro E. D., de Lima Damasceno B.
P. G., Alves P. M., Nonaka C. F. W., Pereira J. V. (2025). Development of citral-based hydrogel
for the treatment of oral candidosis: in vitro and in vivo study. Clin Oral Investig.

[49] Chen Y., Guo B., Zhang Y., Bao X., Li D., Lin J. (2025). Injectable
hypoxia-preconditioned human exfoliated deciduous teeth stem cells
encapsulated within GelMA-AMP microspheres for bone regeneration in
periodontitis. Colloids Surf., B.

